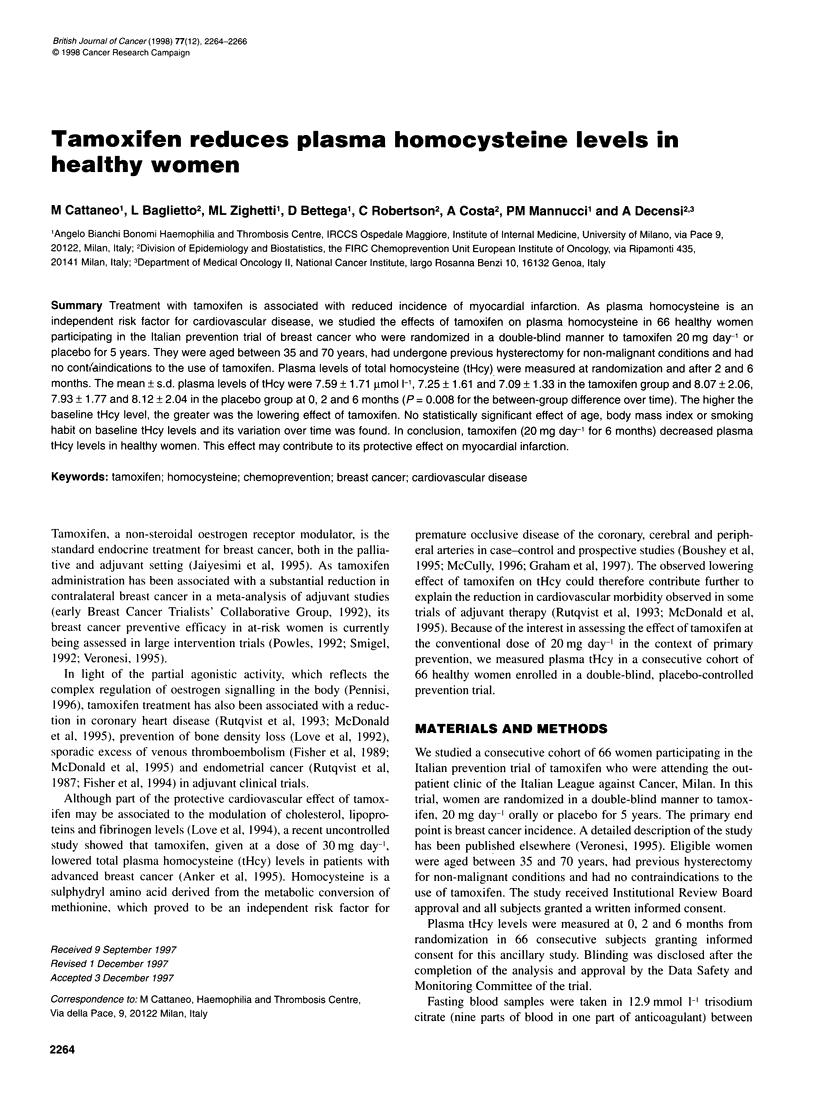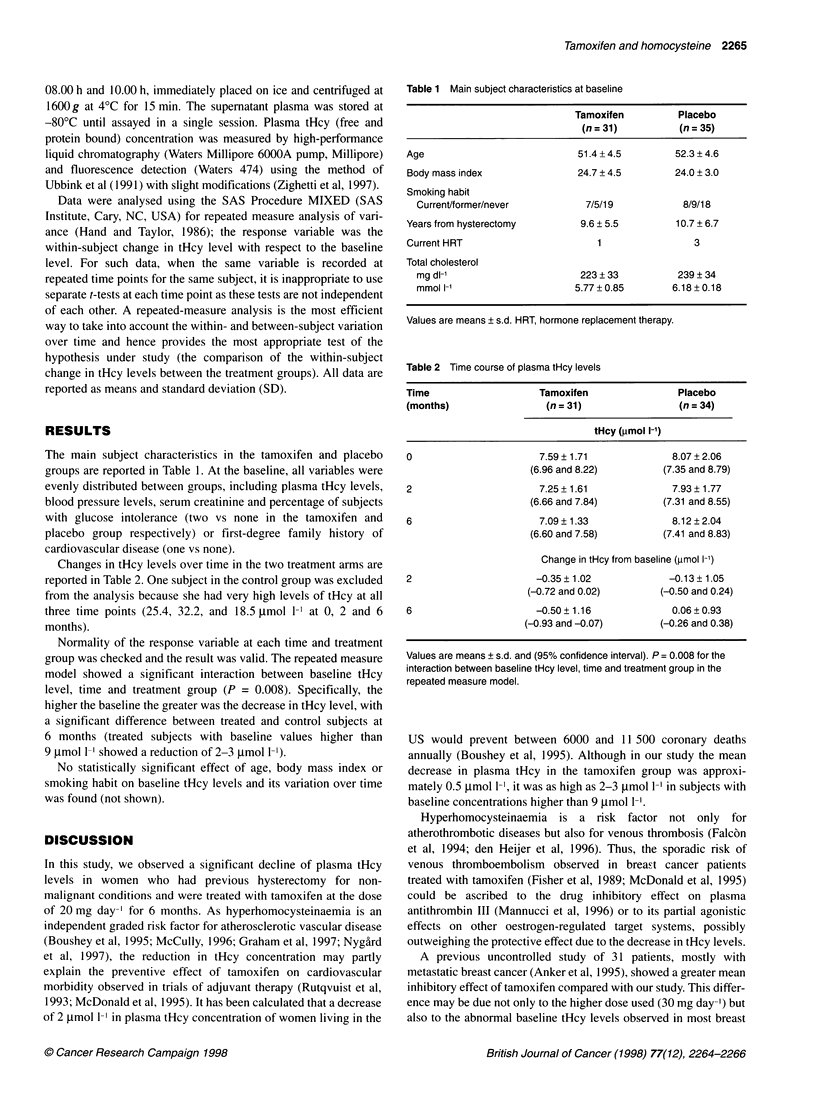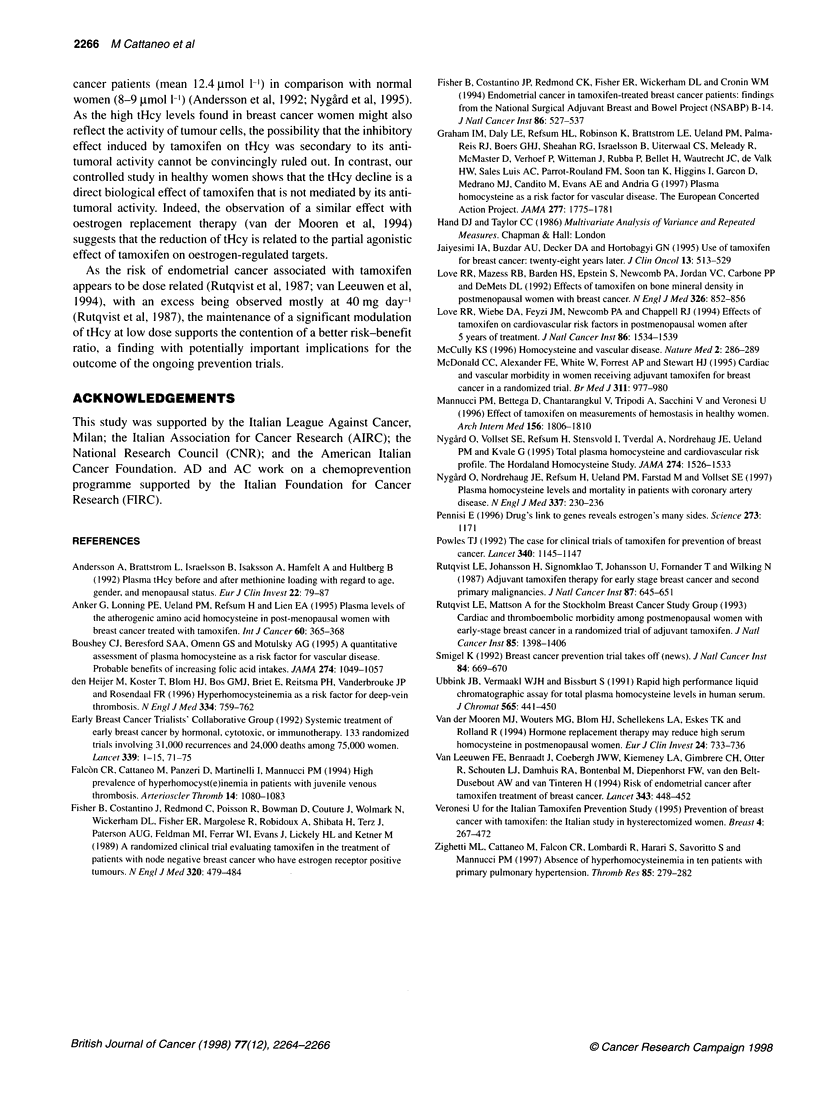# Tamoxifen reduces plasma homocysteine levels in healthy women.

**DOI:** 10.1038/bjc.1998.376

**Published:** 1998-06

**Authors:** M. Cattaneo, L. Baglietto, M. L. Zighetti, D. Bettega, C. Robertson, A. Costa, P. M. Mannucci, A. Decensi

**Affiliations:** Angelo Bianchi Bonomi Haemophilia and Thrombosis Centre, IRCCS Ospedale Maggiore, Institute of Internal Medicine, University of Milano, Milan, Italy.

## Abstract

Treatment with tamoxifen is associated with reduced incidence of myocardial infarction. As plasma homocysteine is an independent risk factor for cardiovascular disease, we studied the effects of tamoxifen on plasma homocysteine in 66 healthy women participating in the Italian prevention trial of breast cancer who were randomized in a double-blind manner to tamoxifen 20 mg day(-1) or placebo for 5 years. They were aged between 35 and 70 years, had undergone previous hysterectomy for non-malignant conditions and had no contraindications to the use of tamoxifen. Plasma levels of total homocysteine (tHcy) were measured at randomization and after 2 and 6 months. The mean +/- s.d. plasma levels of tHcy were 7.59 +/- 1.71 micromol l(-1), 7.25 +/- 1.61 and 7.09 +/- 1.33 in the tamoxifen group and 8.07 +/- 2.06, 7.93 +/- 1.77 and 8.12 +/- 2.04 in the placebo group at 0, 2 and 6 months (P = 0.008 for the between-group difference over time). The higher the baseline tHcy level, the greater was the lowering effect of tamoxifen. No statistically significant effect of age, body mass index or smoking habit on baseline tHcy levels and its variation over time was found. In conclusion, tamoxifen (20 mg day(-1) for 6 months) decreased plasma tHcy levels in healthy women. This effect may contribute to its protective effect on myocardial infarction.


British Journal of Cancer (1998) 77(12), 2264–2266
© 1998 Cancer Research Campaign

### Tamoxifen reduces plasma homocysteine levels in healthy women

**M Cattaneo[1], L Baglietto[2], ML Zighetti[1], D Bettega[1], C Robertson[2], A Costa[2], PM Mannucci[1] and A Decensi[2,3]**

[1]Angelo Bianchi Bonomi Haemophilia and Thrombosis Centre, IRCCS Ospedale Maggiore, Institute of Internal Medicine, University of Milano, via Pace 9, 20122, Milan, Italy; [2]Division of Epidemiology and Biostatistics, the FIRC Chemoprevention Unit European Institute of Oncology, via Ripamonti 435, 20141 Milan, Italy; [3]Department of Medical Oncology II, National Cancer Institute, largo Rosanna Benzi 10, 16132 Genoa, Italy

**Summary** Treatment with tamoxifen is associated with reduced incidence of myocardial infarction. As plasma homocysteine is an independent risk factor for cardiovascular disease, we studied the effects of tamoxifen on plasma homocysteine in 66 healthy women participating in the Italian prevention trial of breast cancer who were randomized in a double-blind manner to tamoxifen 20 mg $day^{-1}$ or placebo for 5 years. They were aged between 35 and 70 years, had undergone previous hysterectomy for non-malignant conditions and had no contraindications to the use of tamoxifen. Plasma levels of total homocysteine (tHcy) were measured at randomization and after 2 and 6 months. The mean ± s.d. plasma levels of tHcy were 7.59 ± 1.71 μmol $l^{-1}$, 7.25 ± 1.61 and 7.09 ± 1.33 in the tamoxifen group and 8.07 ± 2.06, 7.93 ± 1.77 and 8.12 ± 2.04 in the placebo group at 0, 2 and 6 months ($P = 0.008$ for the between-group difference over time). The higher the baseline tHcy level, the greater was the lowering effect of tamoxifen. No statistically significant effect of age, body mass index or smoking habit on baseline tHcy levels and its variation over time was found. In conclusion, tamoxifen (20 mg $day^{-1}$ for 6 months) decreased plasma tHcy levels in healthy women. This effect may contribute to its protective effect on myocardial infarction.

**Keywords:** tamoxifen; homocysteine; chemoprevention; breast cancer; cardiovascular disease

Tamoxifen, a non-steroidal oestrogen receptor modulator, is the standard endocrine treatment for breast cancer, both in the palliative and adjuvant setting (Jaiyesimi et al, 1995). As tamoxifen administration has been associated with a substantial reduction in contralateral breast cancer in a meta-analysis of adjuvant studies (early Breast Cancer Trialists' Collaborative Group, 1992), its breast cancer preventive efficacy in at-risk women is currently being assessed in large intervention trials (Powles, 1992; Smigel, 1992; Veronesi, 1995).

In light of the partial agonistic activity, which reflects the complex regulation of oestrogen signalling in the body (Pennisi, 1996), tamoxifen treatment has also been associated with a reduction in coronary heart disease (Rutqvist et al, 1993; McDonald et al, 1995), prevention of bone density loss (Love et al, 1992), sporadic excess of venous thromboembolism (Fisher et al, 1989; McDonald et al, 1995) and endometrial cancer (Rutqvist et al, 1987; Fisher et al, 1994) in adjuvant clinical trials.

Although part of the protective cardiovascular effect of tamoxifen may be associated to the modulation of cholesterol, lipoproteins and fibrinogen levels (Love et al, 1994), a recent uncontrolled study showed that tamoxifen, given at a dose of 30 mg $day^{-1}$, lowered total plasma homocysteine (tHcy) levels in patients with advanced breast cancer (Anker et al, 1995). Homocysteine is a sulphydryl amino acid derived from the metabolic conversion of methionine, which proved to be an independent risk factor for premature occlusive disease of the coronary, cerebral and peripheral arteries in case–control and prospective studies (Boushey et al, 1995; McCully, 1996; Graham et al, 1997). The observed lowering effect of tamoxifen on tHcy could therefore contribute further to explain the reduction in cardiovascular morbidity observed in some trials of adjuvant therapy (Rutqvist et al, 1993; McDonald et al, 1995). Because of the interest in assessing the effect of tamoxifen at the conventional dose of 20 mg $day^{-1}$ in the context of primary prevention, we measured plasma tHcy in a consecutive cohort of 66 healthy women enrolled in a double-blind, placebo-controlled prevention trial.

*Received 9 September 1997*
*Revised 1 December 1997*
*Accepted 3 December 1997*

*Correspondence to:* M Cattaneo, Haemophilia and Thrombosis Centre, Via della Pace, 9, 20122 Milan, Italy

### MATERIALS AND METHODS

We studied a consecutive cohort of 66 women participating in the Italian prevention trial of tamoxifen who were attending the outpatient clinic of the Italian League against Cancer, Milan. In this trial, women are randomized in a double-blind manner to tamoxifen, 20 mg $day^{-1}$ orally or placebo for 5 years. The primary end point is breast cancer incidence. A detailed description of the study has been published elsewhere (Veronesi, 1995). Eligible women were aged between 35 and 70 years, had previous hysterectomy for non-malignant conditions and had no contraindications to the use of tamoxifen. The study received Institutional Review Board approval and all subjects granted a written informed consent.

Plasma tHcy levels were measured at 0, 2 and 6 months from randomization in 66 consecutive subjects granting informed consent for this ancillary study. Blinding was disclosed after the completion of the analysis and approval by the Data Safety and Monitoring Committee of the trial.

Fasting blood samples were taken in 12.9 mmol $l^{-1}$ trisodium citrate (nine parts of blood in one part of anticoagulant) between

08.00 h and 10.00 h, immediately placed on ice and centrifuged at 1600 *g* at 4°C for 15 min. The supernatant plasma was stored at –80°C until assayed in a single session. Plasma tHcy (free and protein bound) concentration was measured by high-performance liquid chromatography (Waters Millipore 6000A pump, Millipore) and fluorescence detection (Waters 474) using the method of Ubbink et al (1991) with slight modifications (Zighetti et al, 1997).

Data were analysed using the SAS Procedure MIXED (SAS Institute, Cary, NC, USA) for repeated measure analysis of variance (Hand and Taylor, 1986); the response variable was the within-subject change in tHcy level with respect to the baseline level. For such data, when the same variable is recorded at repeated time points for the same subject, it is inappropriate to use separate *t*-tests at each time point as these tests are not independent of each other. A repeated-measure analysis is the most efficient way to take into account the within- and between-subject variation over time and hence provides the most appropriate test of the hypothesis under study (the comparison of the within-subject change in tHcy levels between the treatment groups). All data are reported as means and standard deviation (SD).

### RESULTS

The main subject characteristics in the tamoxifen and placebo groups are reported in Table 1. At the baseline, all variables were evenly distributed between groups, including plasma tHcy levels, blood pressure levels, serum creatinine and percentage of subjects with glucose intolerance (two vs none in the tamoxifen and placebo group respectively) or first-degree family history of cardiovascular disease (one vs none).

Changes in tHcy levels over time in the two treatment arms are reported in Table 2. One subject in the control group was excluded from the analysis because she had very high levels of tHcy at all three time points (25.4, 32.2, and 18.5 µmol $l^{-1}$ at 0, 2 and 6 months).

Normality of the response variable at each time and treatment group was checked and the result was valid. The repeated measure model showed a significant interaction between baseline tHcy level, time and treatment group ($P$ = 0.008). Specifically, the higher the baseline the greater was the decrease in tHcy level, with a significant difference between treated and control subjects at 6 months (treated subjects with baseline values higher than 9 µmol $l^{-1}$ showed a reduction of 2–3 µmol $l^{-1}$).

No statistically significant effect of age, body mass index or smoking habit on baseline tHcy levels and its variation over time was found (not shown).

**Table 1** Main subject characteristics at baseline

| | Tamoxifen ($n$ = 31) | Placebo ($n$ = 35) |
|---|---|---|
| Age | 51.4 ± 4.5 | 52.3 ± 4.6 |
| Body mass index | 24.7 ± 4.5 | 24.0 ± 3.0 |
| Smoking habit | | |
| Current/former/never | 7/5/19 | 8/9/18 |
| Years from hysterectomy | 9.6 ± 5.5 | 10.7 ± 6.7 |
| Current HRT | 1 | 3 |
| Total cholesterol | | |
| mg $dl^{-1}$ | 223 ± 33 | 239 ± 34 |
| mmol $l^{-1}$ | 5.77 ± 0.85 | 6.18 ± 0.18 |

Values are means ± s.d. HRT, hormone replacement therapy.

**Table 2** Time course of plasma tHcy levels

| Time (months) | Tamoxifen ($n$ = 31) | Placebo ($n$ = 34) |
|---|---|---|
| | tHcy (µmol $l^{-1}$) | |
| 0 | 7.59 ± 1.71 (6.96 and 8.22) | 8.07 ± 2.06 (7.35 and 8.79) |
| 2 | 7.25 ± 1.61 (6.66 and 7.84) | 7.93 ± 1.77 (7.31 and 8.55) |
| 6 | 7.09 ± 1.33 (6.60 and 7.58) | 8.12 ± 2.04 (7.41 and 8.83) |
| | Change in tHcy from baseline (µmol $l^{-1}$) | |
| 2 | –0.35 ± 1.02 (–0.72 and 0.02) | –0.13 ± 1.05 (–0.50 and 0.24) |
| 6 | –0.50 ± 1.16 (–0.93 and –0.07) | 0.06 ± 0.93 (–0.26 and 0.38) |

Values are means ± s.d. and (95% confidence interval). $P$ = 0.008 for the interaction between baseline tHcy level, time and treatment group in the repeated measure model.

### DISCUSSION

In this study, we observed a significant decline of plasma tHcy levels in women who had previous hysterectomy for non-malignant conditions and were treated with tamoxifen at the dose of 20 mg $day^{-1}$ for 6 months. As hyperhomocysteinaemia is an independent graded risk factor for atherosclerotic vascular disease (Boushey et al, 1995; McCully, 1996; Graham et al, 1997; Nygård et al, 1997), the reduction in tHcy concentration may partly explain the preventive effect of tamoxifen on cardiovascular morbidity observed in trials of adjuvant therapy (Rutqvuist et al, 1993; McDonald et al, 1995). It has been calculated that a decrease of 2 µmol $l^{-1}$ in plasma tHcy concentration of women living in the US would prevent between 6000 and 11 500 coronary deaths annually (Boushey et al, 1995). Although in our study the mean decrease in plasma tHcy in the tamoxifen group was approximately 0.5 µmol $l^{-1}$, it was as high as 2–3 µmol $l^{-1}$ in subjects with baseline concentrations higher than 9 µmol $l^{-1}$.

Hyperhomocysteinaemia is a risk factor not only for atherothrombotic diseases but also for venous thrombosis (Falcòn et al, 1994; den Heijer et al, 1996). Thus, the sporadic risk of venous thromboembolism observed in breast cancer patients treated with tamoxifen (Fisher et al, 1989; McDonald et al, 1995) could be ascribed to the drug inhibitory effect on plasma antithrombin III (Mannucci et al, 1996) or to its partial agonistic effects on other oestrogen-regulated target systems, possibly outweighing the protective effect due to the decrease in tHcy levels.

A previous uncontrolled study of 31 patients, mostly with metastatic breast cancer (Anker et al, 1995), showed a greater mean inhibitory effect of tamoxifen compared with our study. This difference may be due not only to the higher dose used (30 mg $day^{-1}$) but also to the abnormal baseline tHcy levels observed in most breast

© Cancer Research Campaign 1998

cancer patients (mean 12.4 µmol $l^{-1}$) in comparison with normal women (8–9 µmol $l^{-1}$) (Andersson et al, 1992; Nygård et al, 1995). As the high tHcy levels found in breast cancer women might also reflect the activity of tumour cells, the possibility that the inhibitory effect induced by tamoxifen on tHcy was secondary to its anti-tumoral activity cannot be convincingly ruled out. In contrast, our controlled study in healthy women shows that the tHcy decline is a direct biological effect of tamoxifen that is not mediated by its anti-tumoral activity. Indeed, the observation of a similar effect with oestrogen replacement therapy (van der Mooren et al, 1994) suggests that the reduction of tHcy is related to the partial agonistic effect of tamoxifen on oestrogen-regulated targets.

As the risk of endometrial cancer associated with tamoxifen appears to be dose related (Rutqvist et al, 1987; van Leeuwen et al, 1994), with an excess being observed mostly at 40 mg $day^{-1}$ (Rutqvist et al, 1987), the maintenance of a significant modulation of tHcy at low dose supports the contention of a better risk–benefit ratio, a finding with potentially important implications for the outcome of the ongoing prevention trials.

### ACKNOWLEDGEMENTS

This study was supported by the Italian League Against Cancer, Milan; the Italian Association for Cancer Research (AIRC); the National Research Council (CNR); and the American Italian Cancer Foundation. AD and AC work on a chemoprevention programme supported by the Italian Foundation for Cancer Research (FIRC).

### REFERENCES

Andersson A, Brattstrom L, Israelsson B, Isaksson A, Hamfelt A and Hultberg B (1992) Plasma tHcy before and after methionine loading with regard to age, gender, and menopausal status. *Eur J Clin Invest* **22**: 79–87

Anker G, Lonning PE, Ueland PM, Refsum H and Lien EA (1995) Plasma levels of the atherogenic amino acid homocysteine in post-menopausal women with breast cancer treated with tamoxifen. *Int J Cancer* **60**: 365–368

Boushey CJ, Beresford SAA, Omenn GS and Motulsky AG (1995) A quantitative assessment of plasma homocysteine as a risk factor for vascular disease. Probable benefits of increasing folic acid intakes. *JAMA* **274**: 1049–1057

den Heijer M, Koster T, Blom HJ, Bos GMJ, Briet E, Reitsma PH, Vanderbrouke JP and Rosendaal FR (1996) Hyperhomocysteinemia as a risk factor for deep-vein thrombosis. *N Engl J Med* **334**: 759–762

Early Breast Cancer Trialists' Collaborative Group (1992) Systemic treatment of early breast cancer by hormonal, cytotoxic, or immunotherapy. 133 randomized trials involving 31,000 recurrences and 24,000 deaths among 75,000 women. *Lancet* **339**: 1–15, 71–75

Falcòn CR, Cattaneo M, Panzeri D, Martinelli I, Mannucci PM (1994) High prevalence of hyperhomocyst(e)inemia in patients with juvenile venous thrombosis. *Arterioscler Thromb* **14**: 1080–1083

Fisher B, Costantino J, Redmond C, Poisson R, Bowman D, Couture J, Wolmark N, Wickerham DL, Fisher ER, Margolese R, Robidoux A, Shibata H, Terz J, Paterson AUG, Feldman MI, Ferrar WI, Evans J, Lickely HL and Ketner M (1989) A randomized clinical trial evaluating tamoxifen in the treatment of patients with node negative breast cancer who have estrogen receptor positive tumours. *N Engl J Med* **320**: 479–484

Fisher B, Costantino JP, Redmond CK, Fisher ER, Wickerham DL and Cronin WM (1994) Endometrial cancer in tamoxifen-treated breast cancer patients: findings from the National Surgical Adjuvant Breast and Bowel Project (NSABP) B-14. *J Natl Cancer Inst* **86**: 527–537

Graham IM, Daly LE, Refsum HL, Robinson K, Brattstrom LE, Ueland PM, Palma-Reis RJ, Boers GHJ, Sheahan RG, Israelsson B, Uiterwaal CS, Meleady R, McMaster D, Verhoef P, Witteman J, Rubba P, Bellet H, Wautrecht JC, de Valk HW, Sales Luis AC, Parrot-Rouland FM, Soon tan K, Higgins I, Garcon D, Medrano MJ, Candito M, Evans AE and Andria G (1997) Plasma homocysteine as a risk factor for vascular disease. The European Concerted Action Project. *JAMA* **277**: 1775–1781

Hand DJ and Taylor CC (1986) *Multivariate Analysis of Variance and Repeated Measures*. Chapman & Hall: London

Jaiyesimi IA, Buzdar AU, Decker DA and Hortobagyi GN (1995) Use of tamoxifen for breast cancer: twenty-eight years later. *J Clin Oncol* **13**: 513–529

Love RR, Mazess RB, Barden HS, Epstein S, Newcomb PA, Jordan VC, Carbone PP and DeMets DL (1992) Effects of tamoxifen on bone mineral density in postmenopausal women with breast cancer. *N Engl J Med* **326**: 852–856

Love RR, Wiebe DA, Feyzi JM, Newcomb PA and Chappell RJ (1994) Effects of tamoxifen on cardiovascular risk factors in postmenopausal women after 5 years of treatment. *J Natl Cancer Inst* **86**: 1534–1539

McCully KS (1996) Homocysteine and vascular disease. *Nature Med* **2**: 286–289

McDonald CC, Alexander FE, White W, Forrest AP and Stewart HJ (1995) Cardiac and vascular morbidity in women receiving adjuvant tamoxifen for breast cancer in a randomized trial. *Br Med J* **311**: 977–980

Mannucci PM, Bettega D, Chantarangkul V, Tripodi A, Sacchini V and Veronesi U (1996) Effect of tamoxifen on measurements of hemostasis in healthy women. *Arch Intern Med* **156**: 1806–1810

Nygård O, Vollset SE, Refsum H, Stensvold I, Tverdal A, Nordrehaug JE, Ueland PM and Kvale G (1995) Total plasma homocysteine and cardiovascular risk profile. The Hordaland Homocysteine Study. *JAMA* **274**: 1526–1533

Nygård O, Nordrehaug JE, Refsum H, Ueland PM, Farstad M and Vollset SE (1997) Plasma homocysteine levels and mortality in patients with coronary artery disease. *N Engl J Med* **337**: 230–236

Pennisi E (1996) Drug's link to genes reveals estrogen's many sides. *Science* **273**: 1171

Powles TJ (1992) The case for clinical trials of tamoxifen for prevention of breast cancer. *Lancet* **340**: 1145–1147

Rutqvist LE, Johansson H, Signomklao T, Johansson U, Fornander T and Wilking N (1987) Adjuvant tamoxifen therapy for early stage breast cancer and second primary malignancies. *J Natl Cancer Inst* **87**: 645–651

Rutqvist LE, Mattson A for the Stockholm Breast Cancer Study Group (1993) Cardiac and thromboembolic morbidity among postmenopausal women with early-stage breast cancer in a randomized trial of adjuvant tamoxifen. *J Natl Cancer Inst* **85**: 1398–1406

Smigel K (1992) Breast cancer prevention trial takes off (news). *J Natl Cancer Inst* **84**: 669–670

Ubbink JB, Vermaakl WJH and Bissburt S (1991) Rapid high performance liquid chromatographic assay for total plasma homocysteine levels in human serum. *J Chromat* **565**: 441–450

Van der Mooren MJ, Wouters MG, Blom HJ, Schellekens LA, Eskes TK and Rolland R (1994) Hormone replacement therapy may reduce high serum homocysteine in postmenopausal women. *Eur J Clin Invest* **24**: 733–736

Van Leeuwen FE, Benraadt J, Coebergh JWW, Kiemeney LA, Gimbrere CH, Otter R, Schouten LJ, Damhuis RA, Bontenbal M, Diepenhorst FW, van den Belt-Dusebout AW and van Tinteren H (1994) Risk of endometrial cancer after tamoxifen treatment of breast cancer. *Lancet* **343**: 448–452

Veronesi U for the Italian Tamoxifen Prevention Study (1995) Prevention of breast cancer with tamoxifen: the Italian study in hysterectomized women. *Breast* **4**: 267–472

Zighetti ML, Cattaneo M, Falcon CR, Lombardi R, Harari S, Savoritto S and Mannucci PM (1997) Absence of hyperhomocysteinemia in ten patients with primary pulmonary hypertension. *Thromb Res* **85**: 279–282

© Cancer Research Campaign 1998